# Transcriptional profiles in the mouse amygdala after a cognitive judgment bias test largely depend on the genotype

**DOI:** 10.3389/fnmol.2022.1025389

**Published:** 2022-10-24

**Authors:** Marisol Herrera-Rivero, Lena Bohn, Anika Witten, Kay Jüngling, Sylvia Kaiser, S. Helene Richter, Monika Stoll, Norbert Sachser

**Affiliations:** ^1^Department of Genetic Epidemiology, Institute of Human Genetics, University of Münster, Münster, Germany; ^2^Department of Psychiatry, University of Münster, Münster, Germany; ^3^Department of Behavioral Biology, Institute of Neuro- and Behavioral Biology, University of Münster, Münster, Germany; ^4^Münster Graduate School of Evolution, University of Münster, Münster, Germany; ^5^Core Facility Genomics, Medical Faculty, University of Münster, Münster, Germany; ^6^Institute of Physiology I, University of Münster, Münster, Germany

**Keywords:** amygdala, differential expression, genotype, environment, cognitive bias

## Abstract

**Background:** The amygdala is crucial for emotional cognitive processing. Affective or emotional states can bias cognitive processes, including attention, memory, and decision-making. This can result in optimistic or pessimistic behaviors that are partially driven by the activation of the amygdala. The resulting emotional cognitive bias is a common feature of anxiety and mood disorders, both of which are interactively influenced by genetic and environmental factors. It is also known that emotional cognitive biases can be influenced by environmental factors. However, little is known about the effects of genetics and/or gene-environment interactions on emotional cognitive biases. We investigated the effects of the genetic background and environmental enrichment on the transcriptional profiles of the mouse amygdala following a well-established cognitive bias test.

**Methods:** Twenty-four female C57BL/6J and B6D2F1N mice were housed either in standard (control) conditions or in an enriched environment. After appropriate training, the cognitive bias test was performed on 19 mice that satisfactorily completed the training scheme to assess their responses to ambiguous cues. This allowed us to calculate an “optimism score” for each mouse. Subsequently, we dissected the anterior and posterior portions of the amygdala to perform RNA-sequencing for differential expression and other statistical analyses.

**Results:** In general, we found only minor changes in the amygdala’s transcriptome associated with the levels of optimism in our mice. In contrast, we observed wide molecular effects of the genetic background in both housing environments. The C57BL/6J animals showed more transcriptional changes in response to enriched environments than the B6D2F1N mice. We also generally found more dysregulated genes in the posterior than in the anterior portion of the amygdala. Gene set overrepresentation analyses consistently implicated cellular metabolic responses and immune processes in the differences observed between mouse strains, while processes favoring neurogenesis and neurotransmission were implicated in the responses to environmental enrichment. In a correlation analysis, lipid metabolism in the anterior amygdala was suggested to influence the levels of optimism.

**Conclusions:** Our observations underscore the importance of selecting appropriate animal models when performing molecular studies of affective conditions or emotional states, and suggest an important role of immune and stress responses in the genetic component of emotion regulation.

## Background

The amygdala, as part of the limbic system, is crucially involved in the regulation of emotional responses, including the emotional perception and encoding of environmental stimuli, as well as emotional memory, decision-making, motivation, and behavior (Ramasubbu et al., 2014). With a complex structure, the amygdala is functionally connected to extensive cortical-subcortical-limbic circuits. Impairments in the functional connectivity of the amygdala with other brain structures might result from stress-induced hyperactivity of the amygdala (Zhang et al., 2018). Impairments in functional connections, such as those of the anterior portion of the amygdala with the anterior cingulate cortex and the prefrontal cortex, as well as of the posterior portion of the amygdala with the hippocampus, are features of neuropsychiatric diseases, including anxiety, depression, and bipolar disorder, which possess strong emotional behavioral and cognitive components (Ramasubbu et al., 2014; Yang and Wang, 2017; Li et al., 2018).

For an individual’s fitness, survival, and well-being, the ability to respond to environmental cues with appropriate behaviors is crucial. However, when external cues are ambiguous, decision-making and behavior in response to these are biased by the individual’s internal state. Individuals in a putatively positive internal state tend to interpret ambiguous cues more optimistically, expecting positive outcomes (e.g., rewards), while individuals in a putatively negative state tend to interpret the same cues more pessimistically (Paul et al., 2005; Mendl et al., 2010; Rygula et al., 2013). In humans, emotional cognitive bias occurs in anxiety and mood disorders and has been partially attributed to changes in the activity of the amygdala (Drevets, 2003; Victor et al., 2010; Ramasubbu et al., 2014; Yang and Wang, 2017; Zhang et al., 2018).

Emotional cognitive bias can be assessed in humans and animals through the individual’s behavioral responses toward ambiguous cues (Rohrbacher and Reinecke, 2014; Roelofs et al., 2016). In this manner, previous studies have established that environmental factors influence individual differences in emotional cognitive bias. In animal models of depression, for example, early life adversity was associated with increased vulnerability to negative bias and impaired ability to learn reward value (Stuart et al., 2019), while environmental enrichment was associated with increased positive bias (Richter et al., 2012). Moreover, most animal welfare studies have shown that the immediate social and physical environment plays a role in shaping an individual’s emotional cognitive bias. For example, enriched or impoverished housing conditions, access or deprivation of social partners, and varying levels of perceived predation pressure are factors known to influence emotional cognitive bias (Bateson and Matheson, 2007; Bateson et al., 2011; Brydges et al., 2011; Douglas et al., 2012; Richter et al., 2012; Daros et al., 2014; Bethell and Koyama, 2015; Bučková et al., 2019).

Mood disorders are under the combined influence of both genetic and environmental factors (Emamzadeh and Surguchov, 2018). Despite the relatively high heritability of mood disorders (Dalby et al., 2022; Hara et al., 2022; Nguyen et al., 2022) associated with emotional cognitive bias, studies of the genetic factors and molecular mechanisms underlying optimistic and pessimistic biases are lacking. Here, we used two mouse strains in either standard or enriched housing conditions to study: (1) the effects of the genetic background and environmental conditions on emotional cognitive bias; and (2) the link between emotional cognitive bias and the function of the amygdala. We hypothesized that: (1) both the genetic and environmental background interactively shape emotional cognitive bias in mice; and (2) differences in emotional cognitive biases are linked to differential gene expression in the amygdala. We show that mice from different strains possess differential transcriptional patterns regardless of the housing environment or potential emotional cognitive bias, which suggests that genetics played a major role in the function of the amygdala in our study.

## Methods

### Experimental animals

We used 24 focal mice in total, consisting of 12 female C57BL/6J and 12 female B6D2F1N mice. The black coat of these strains permits blinded experimentation. Moreover, both of them are capable of learning our behavioral paradigms (Bračić et al., 2022) and have been reported to express behavioral differences (von Kortzfleisch et al., 2020), characteristics that were important in our experimental settings. Because male mice are more likely to become aggressive toward their cage partners (Van Loo et al., 2003), we chose to use only female mice in this study. All mice were purchased from a professional breeder (Charles River Laboratories, Research Models and Services, Germany GmbH, Sulzfeld, Germany) at the age of 4 weeks. All focal mice were co-housed in the same strain-same sex groups of three individuals per cage (Makrolon cages type III, 38 × 23 × 15 cm^3^). For individual identification, partial ear punches were applied upon arrival. All cages were equipped with wood shavings as bedding material (Allspan, Höveler GmbH and Co. KG, Langenfeld, Germany), a semi-transparent red plastic shelter (11.1 × 11.1 × 5.5 cm^3^, Tecniplast Deutschland GmbH, Hohenpeißenberg, Germany), a semi-transparent red handling tunnel (length: 98.55 mm, diameter: 50.8 mm, ZOONLAB GmbH, Castrop-Rauxel, Germany), a wooden gnawing stick, and a paper towel. The housing rooms were kept at a temperature of approximately 23°C and relative humidity of about 50%, with a reversed light/dark cycle of 12:12 h, with the lights off from 08:00 h to 20:00 h. Water and food were provided *ad libitum* until the onset of the experimental phase. After the experimental phase started, a restrictive feeding regime was provided, i.e., animals received food once per day to maintain 90%–95% of their *ad libitum* feeding weights. This food restriction scheme had the purpose to increase the motivation to work for food rewards while avoiding a negative impact on welfare (Feige-Diller et al., 2020). Body weights were monitored on a daily basis using a digital scale (resolution: 0.1 g; KERN CM 150-1N pocket balance, KERN and Sohn GmbH, Balingen, Germany). Whenever necessary, the tunnel handling method was applied to transfer mice to their target location (Hurst and West, 2010).

### Experimental design

**Figure 1:**
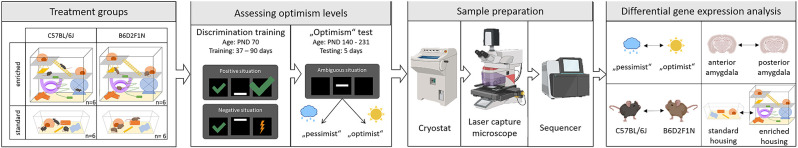
Experimental design. Adapted from Bračić et al. (2022); using templates from BioRender (https:app.biorender.com/biorender-templates: mouse-anterior2, [standing, sitting, grooming, running, scratching cheek, and lateral 1-, lightning bolt 1, cloud 1, raindrop, sun, cryostat, laser microdissection microscope, Sequencer-(Illumina NextSeq 500-, and mouse brain -coronal, thalamus and hippocampus and coronal, thalamus and hippocampus 2)].

To test for an effect of genotype and environment on emotional cognitive bias in mice, we used a two-by-two full factorial design, where mice from both strains (C57BL/6J and B6D2F1N) were randomly assigned to either standard or enriched (with access to a playground) housing conditions. Therefore, four experimental groups were included in the study. The environmental treatment commenced on the post-natal day (PND) 77 and continued until the end of the experiment. Emotional cognitive bias was assessed using an active choice (Go/Go-task) cognitive judgment bias (CJB) test. As the CJB test requires prior discrimination training, we began training the animals on PND 70. Training duration, though, was dependent on the learning speed of each mouse (37–90 days). Performing the CJB test itself took 5 days. Five mice that did not fulfill the learning criterion were excluded. Therefore, 19 mice that completed the CBJ test were euthanized to dissect the anterior and posterior portions of the amygdala for transcriptome analyses (Figure 1).

### Housing environments

While half of the mice from each strain were assigned to the standard housing condition mentioned above, the other half were assigned to the enriched housing condition where the animals had daily access to a highly enriched cage (50 × 32 × 52 cm^3^), in a manner of “playgrounds”. Each of these cages was connected to one of the playgrounds for one hour per day, and mice were allowed to freely move between their home cage and the respective playground. For a more detailed description, please refer to (Bračić et al., 2022). To control for potential handling effects, mice from the standard housing condition received a mock treatment, where their standard cages were taken out of the racks and placed on a work bench next to their racks within the housing room. After one hour, mice from both treatment groups were fed and placed back into the racks.

### Touchscreen training and cognitive judgment bias (CJB) testing

Emotional cognitive bias was assessed through CJB testing using a touchscreen-based active choice (go/go) task (Bussey-Saksida Mouse Touch Screen Chambers, Model 80614, Campden Instruments Ltd., Loughborough, United Kingdom). This test requires prior training so that the animals learn first how to operate the touchscreen chambers and then to respond discriminatively towards two reference cues (Bračić et al., 2022).

Each training chamber was equipped with a touchscreen, a reward dispenser, a light, and a speaker. The touchscreen was divided into three equally sized squares. The middle square presented the mice with a cue, to which the mouse could respond by touching either of the outer two squares. A white horizontal bar displayed at different positions and servings of diluted sweet condensed milk (Nestlé “Milchmädchen gezuckerte Kondensmilch”; diluted 1:4 in tap water) were used as cues and rewards, respectively.

During the touchscreen training, mice learned to discriminate between the positive reference cue (bar position: down) and the negative reference cue (bar position: up). When the positive reference cue was displayed, the mice were rewarded with 12 μl diluted condensed milk for touching the correct side and 4 μl for touching the wrong side. When the negative reference cue was displayed, the mice were rewarded with 4 μl for touching the correct side, or received a mild punishment (5-s timeout with lights on) for touching the wrong side. For a more detailed training scheme, please refer to Bračić et al. (2022).

Upon CJB testing, mice were classified as either “optimistic”, when they showed a positive bias, or “pessimistic”, when they showed a negative bias. This classification was achieved by assessing their responses to unknown, intermediate cues. For this, we displayed the bar in three intermediate positions: middle (the bar was between the lower reference cue and the upper reference cue), near negative (the bar was between the middle cue and the upper reference cue), and near positive (the bar was between the middle cue and the lower reference cue). When a mouse responded to this intermediate cue in the same manner as it would toward the positive cue, an optimistic expectation was assumed. Contrarily, when the response was the same as towards the negative cue, a pessimistic expectation was assumed. Subsequently, the observed responses were used to quantitatively characterize the level of optimistic bias by calculating a score using the following formula:


$$optimism\;level\text{ = }\frac{N\;choice\;(optimistic)-N\;choice\;(pessimistic)}{N\;choice\;(optimistic+pessimistic)}$$

### Tissue sampling

Three days after the last CJB test, mice were anesthetized using 2.5% isoflurane in oxygen and decapitated. Brains were immediately recovered and frozen on dry ice. Afterward, brains were cut with a cryostat (Leica CM30505) into 16–18 μm thick slices, aiming at the posterior and anterior parts of the basolateral nucleus of the amygdala (−0.58 to −0.94 and −2.06 to −2.3 relative to bregma, respectively), here on referred to as posterior and anterior (portions of the) amygdala. Brain slices were picked up using membrane slides (Carl Zeiss^TM^ Membrane Slides by Thermo Fisher Scientific Inc.) previously exposed to UV radiation, and fixated for 15 min in 47°C before staining with eosin red (8 min) and methylene blue (3 min). Cells from the anterior and posterior amygdala were then collected using a laser capture microscope (P.A.L.M. Microlaser Technologie, PALM^®^ MicroBeam by Zeiss), following Sangha et al. (2012).

### RNA extraction, library preparation, and sequencing

Thirty-seven tissue samples from 19 animals (Table 1) were used for the analysis of their transcriptomes *via* RNA-sequencing (RNA-seq). Total RNA was isolated using the RNeasy Micro Kit (Qiagen), followed by a DNase digestion step. Library preparation was carried out upon mRNA enrichment with the NEBNext Single Cell/Low Input RNA Library Prep Kit for Illumina (New England BioLabs). Single read sequencing took place on a NextSeq 2000 System (Illumina), using the corresponding NextSeq 2000 P3 v3 chemistry, with a read length of 72 base pairs. The integrity of the RNA and the quality of the library were assessed using a TapeStation 4200 (Agilent).

**Table 1:** Sample composition.

Sample description	Total mice	Total tissues	Anterior amygdala	Posterior amygdala
N	19	37	18	19
Paired samples	19	36	18	18
C57BL/6J strain	8	15	7	8
B6D2F1N strain	11	22	11	11
Standard housing	9	18	9	9
Enriched environment (playground)	10	19	9	10
C57BL/6J in standard	4	8	4	4
B6D2F1N in standard	5	10	5	5
C57BL/6J in playground	4	7	3	4
B6D2F1N in playground	6	12	6	6
Optimist (high score_M)	4	8	4	4
Pessimist (low score_M)	5	9	4	5

### Data pre-processing

Using a molecular barcode, the data was automatically demultiplexed using the Illumina bcl2fastq2 Conversion Software v2.20. FastQ files underwent two rounds of quality control, pre-trimming, and post-alignment, using Fast Q20 v0.11.7 (Andrews, 2010). Removal of Illumina adapters and low-quality sequences was performed with Trimmomatic v0.38 (Bolger et al., 2014). Reads of length <15 bases, as well as leading and/or trailing bases with quality <3 or no base call, and bases with average quality <15 in a 4-base sliding window were removed. Alignment was performed with HISAT2 v2.1.0 (Kim et al., 2019) using the mouse genome assembly mm10 (*Mus musculus*, GRCm38). Mapped reads (primary alignments) were sorted by read name using SAMtools v1.8 (Li et al., 2009), and read counts were calculated with HTSeq v0.11.2 (Anders et al., 2015).

### Differential expression and gene set overrepresentation analyses

Differential expression was assessed using the R package DESeq2 (Love et al., 2014). Transcriptional changes between the anterior and posterior portions of the amygdala were tested separately for B6D2F1N and C57BL/6J mice using the likelihood ratio test (LRT) with a paired design. Transcriptional differences between the B6D2F1N and C57BL/6J strains, stratified by housing environment, as well as between the standard and enriched housing conditions, stratified by strain, were performed using the Wald test. To test for transcriptional changes related to the mice’s optimism score, we adopted an extremes approach in which animals with the most negative bias, i.e., the lowest optimism score (score_*M* ≤ −0.6, *n*_Anterior_ = 4, *n*_Posterior_ = 5), were compared against those with the most positive bias, i.e., the highest optimism score (score_*M* = 0.2, *n* = 4). These tests were adjusted for the different strains and housing environments using LRT. Raw read counts were filtered to remove genes with less than 10 counts prior to analysis. All p-values were corrected for multiple comparisons according to the Benjamini-Hochberg method. Genes were considered differentially expressed when adjusted *p* ≤ 0.05.

To provide a biological context to these findings, each list of differentially expressed genes (DEGs) was subjected to gene set overrepresentation analysis using the web tool g:GOSt from g:Profiler (Raudvere et al., 2019). All gene ontology (GO) and pathways gene set categories available for *Mus musculus* within this tool (GO: biological process, BP, molecular function, MF, cellular component, CC; biological pathways: KEGG, Reactome, Wikipathways), with the exception of GO terms that have been annotated electronically, were retrieved. Gene sets were considered overrepresented following a hypergeometric test within annotated genes that was corrected for multiple comparisons using the Benjamini-Hochberg method (adjusted *p* < 0.05).

### Statistical analyses

Comparisons between strains and housing environments for age, training duration, and optimism scores were performed using analysis of variance (ANOVA) followed by the Tukey honestly significant difference (HSD) *post-hoc* test, binomial regression or a paired *t*-test as appropriate. Two animals within the B6D2F1N strain housed in the standard (control) condition were identified as outliers with relatively lower training duration and negative bias, respectively, and were therefore excluded from the statistical comparisons of these phenotypes. Nevertheless, no reason to exclude them from the differential expression analyses was found. Correlations between optimism scores and levels of over 15,500 amygdala transcripts with recognized gene symbols were tested using the Pearson method with adjustments for strain and environment as well as false discovery rate (FDR) correction. All statistical analyses were performed in the statistical software R.

## Results

### Effects of genotype and environment on behavior

**Figure 2:**
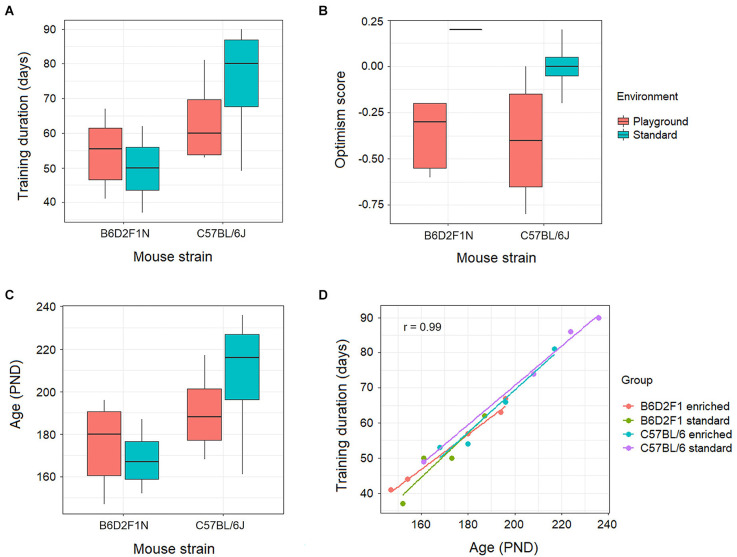
Behavioral effects of genotype (C57BL/6J or B6D2F1N strains) and environment (standard housing conditions or enriched in the form of playground) on **(A)** training duration, **(B)** optimism score, and **(C)** age were not statistically significant (*p* > 0.05). **(D)** Training duration positively correlated (*p* = 2.64 × 10^−14^, *r* = 0.99) with age in all genotypes and environments.

The training duration, measured in days, appeared to be higher for the mice of the C57BL/6J strain, particularly in the standard housing, than for the B6D2F1N animals (Figure 2A). Nevertheless, this did not reach statistical significance (*p* = 0.055). Moreover, our optimism scores did not show associations with genotypes and/or housing environments (*p* = 0.213; Figure 2B), nor correlated with the levels of gene transcripts in either portion of the amygdala at FDR < 0.05. At FDR < 0.25, however, we found 13 gene transcripts (*Kifc2*, *Csta1*, *Asb12*, *Amhr2*, *Akr1b7*, *Tsen54*, *Cyp2e1*, *Mgarp*, *Ptx3*, *BC034090*, *Pcyt2*, *Olah*, and *Idua*) in the anterior portion of the amygdala that negatively correlated with optimism scores (*p* < 0.001, correlation coefficient: −0.8 to −0.86). These were overrepresented (adjusted *p* < 0.05) in lipid metabolic pathways (e.g., Reactome metabolism of lipids, GO lipid hydroxylation).

As the mice of the C57BL/6J strain took longer to learn, these animals ended up being older (Figures 2C,D) than the B6D2F1N mice. However, because this was not a statistically significant difference (*p* = 0.112), the statistical analyses of the transcriptomes were not adjusted for age or training duration.

### Transcriptional differences between the anterior and posterior amygdala

**Figure 3:**
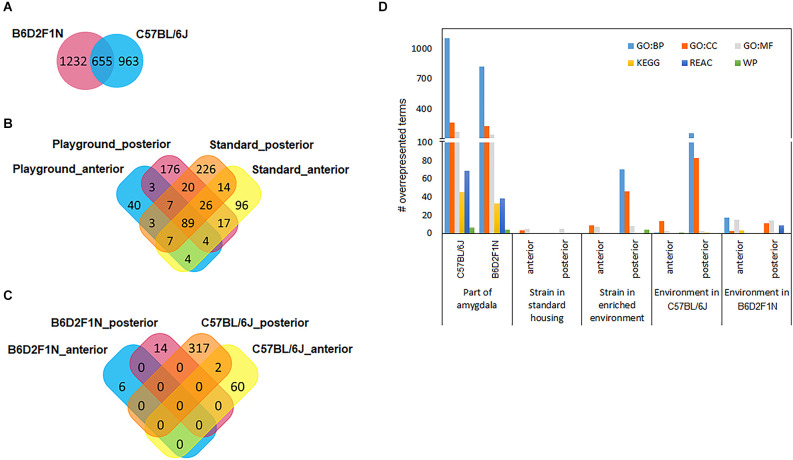
Venn diagrams show how differentially expressed gene transcripts overlap between statistical comparisons performed for **(A)** amygdala regions, **(B)** genotypes, and **(C)** environments. **(D)** Large numbers of functional gene sets were found when comparing portions of the amygdala. GO, gene ontology; BP, biological process; CC, cellular component; MF, molecular function; KEGG, pathways from the Kyoto Encyclopedia of Genes and Genomes; REAC, Reactome pathways; WP, WikiPathways.

Relatively large numbers of gene transcripts were differentially expressed between the anterior and posterior portions of the amygdala in both mouse strains (C57BL/6J: 1618, B6D2F1N: 1887; Supplementary Tables 1,2). About 35%–40% (655) of these overlapped between strains (Figure 3A). A list of the top 10 DEGs for each strain can be found in Table 2. In general, the overlapping DEGs, from which *Bmp3* (*p*_C57BL/6J_ = 1.40 × 10^−49^, *p*_B6D2F1N_ = 5.72 × 10^-28^) appeared among the top 10 findings, functionally implicated a vast number of GO and pathway gene sets (C57BL/6J: 2198, B6D2F1N: 1871; Figure 3D) participating in the development and function of brain cells and in the information exchange within the nervous system by directly affecting neuro- and gliogenesis, neurotransmission, and signaling (Supplementary Table 3). Specific GO terms overrepresented in C57BL/6J mice were overall biased towards regulatory processes, such as secretion and molecular transport. Moreover, the Reactome pathways specifically overrepresented in these mice highlighted cellular metabolism and immune responses, whereas those terms that were specific for B6D2F1N mice appeared to be generally more biased towards metabolic stress responses (Supplementary Table 3).

**Table 2:** Top 10 differentially expressed genes between the anterior and posterior portions of the amygdala in C57BL/6J and B6D2F1N mouse strains.

Symbol	LFC	SE	adj.p	Overlap
**C57BL/6J**				
*Oprk1*	−4.275	0.181	4.89E-91	*n*
*Npsr1*	−5.310	0.243	7.83E-71	y
*Bmp3*	−3.136	0.183	1.49E-49	y
*Vegfd*	−3.686	0.212	8.76E-47	y
*Tmem212*	7.773	0.649	1.70E-44	y
*Kera*	10.242	0.856	3.49E-42	*n*
*Dkkl1*	−3.697	0.238	7.44E-37	y
*Prss23*	−3.103	0.210	1.72E-36	*n*
*Sstr2*	−2.284	0.166	3.20E-34	y
*Dpy19l1*	−1.729	0.126	8.78E-34	y
**B6D2F1N**				
*Xist*	0.532	0.198	1.68E-40	*n*
*Bmp3*	−2.998	0.245	5.72E-28	y
*Krt18*	8.722	0.747	1.68E-20	y
*Steap1*	5.313	0.569	3.56E-19	*n*
*Tspan5*	−1.209	0.132	9.50E-17	y
*Capn11*	0.606	0.521	2.78E-16	*n*
*Olig3*	7.660	0.831	4.79E-16	y
*Nek5*	6.630	0.700	9.73E-16	y
*Rtn4*	−1.312	0.137	3.50E-15	y
*Nrep*	−1.540	0.158	4.59E-15	y

*LFC, log2 fold change; SE, standard error; adj.p, Benjamini-Hochberg-adjusted *p*-value*.

**Table 3:** Top genes differentially expressed in all amygdala regions and housing environments when comparing B6D2F1N and C57BL/6J mice.

Symbol	st.ant.		st.post.		pg.ant.		pg.post.	
	LFC	adj.p	LFC	adj.p	LFC	adj.p	LFC	adj.p
*Cap1*	2.325	6.07E-27	2.81	2.45E-27	2.71	1.21E-39	2.08	1.40E-21
*Cramp1l*	2.026	5.22E-18	2.01	6.62E-19	1.53	2.15E-09	1.46	1.62E-11
*Gabra2*	1.696	2.16E-09	1.49	2.27E-02	1.97	5.66E-04	2.26	2.60E-10
*Gm11273*	2.340	1.50E-04	3.01	1.78E-06	2.69	2.02E-02	2.24	7.09E-03
*Hmga1-rs1*	4.884	5.96E-03	5.51	2.55E-04	5.07	8.23E-03	7.39	5.32E-06
*Lsm12*	1.425	3.52E-05	1.32	1.35E-06	1.40	3.89E-02	1.04	1.13E-02
*Luc7l*	1.771	1.10E-15	1.31	1.20E-09	1.71	2.44E-08	1.21	5.75E-08
*Mark3*	1.020	8.39E-05	0.74	3.14E-03	1.11	1.42E-03	1.17	6.61E-06
*Myo19*	1.799	4.02E-03	2.63	1.03E-14	1.53	7.27E-03	1.67	1.15E-05
*Serpina3m*	6.294	1.16E-07	8.03	1.95E-08	8.10	3.77E-06	5.67	1.17E-07
*Tmem87a*	2.934	1.15E-22	3.04	5.37E-19	3.19	1.23E-12	3.15	2.35E-35
*Traf3ip1*	0.927	6.45E-03	1.48	1.07E-05	1.45	1.80E-03	1.40	1.19E-05
*Wdfy1*	1.690	8.98E-12	1.12	1.75E-05	1.15	2.39E-03	1.38	1.14E-09

*st, standard housing; pg, playground (enriched environment); ant, anterior amygdala; post, posterior amygdala; LFC, log2 fold change; adj.p, Benjamini-Hochberg-adjusted *p*-value*.

### Effects of genetics on the amygdala’s transcriptome

To study the effects of genetic background, we compared the transcriptomes of the anterior and posterior amygdala of mice of the B6D2F1N and C57BL/6J strains housed in standard or enriched environments. In general, we observed larger numbers of DEGs in mice kept in standard housing than in those kept in the enriched environment, as well as in the posterior amygdala with respect to the anterior portion. For mice kept in standard housing, we found 257 DEGs in the anterior amygdala (Supplementary Table 4) and 392 DEGs in the posterior amygdala (Supplementary Table 5). From these, 96 (37.4%) and 226 (57.7%) genes were specifically dysregulated in the anterior and posterior amygdala, respectively (Figure 3B). In the enriched environment, 157 and 342 genes were dysregulated in the anterior and posterior amygdala, being 40 (25.5%) and 176 (51.5%) specifically dysregulated, respectively (Supplementary Tables 6, 7, Figure 3B). In total, 89 DEGs overlapped between all four comparisons (Figure 3B) and importantly represented the most significant findings including, for example, *Cap1*, *Cramp1l*, and *Tmem87a* (Table 3). Moreover, with the exception of the comparison of the posterior amygdala in an enriched environment, only a few GO terms showed overrepresentation in these genotype comparisons (Figure 3D). Overall, while DEGs in the anterior amygdala were overrepresented in terms related to immune responses, those in the posterior amygdala were overrepresented in functional terms related to intercellular communication, neurotransmission, and cellular stress responses (Supplementary Table 8).

### Effects of housing environment on the amygdala’s transcriptome

We also tested whether the mice of the C57BL/6J and B6D2F1N strains presented transcriptional changes in the amygdala depending on the environment in which they were kept by comparing the enriched and standard housing conditions separately for each genotype and amygdala portion. Overall, we found larger differences in C57BL/6J mice than in the B6D2F1N strain, as well as in the posterior than in the anterior portion of the amygdala (#DEGs C57BL/6J_ant_: 62, C57BL/6J_post_: 319, B6D2F1N_ant_: 6, B6D2F1N_post_: 14; Supplementary Tables 9–11). Unlike in the cases of the comparisons between strains and amygdala portions, the transcriptional differences that we observed here were virtually unique (Figure 3C). Only two genes, *Cplx2* (p_ant_ = 0.01117, p_post_ = 0.00015) and *B230323A14Rik* (p_ant_ = 0.01195, p_post_ = 0.03927), were dysregulated in both regions of the amygdala in the C57BL/6J genotype. Up to 10 of the most significant DEGs found for each comparison are presented in Table 4. Overrepresented GO and pathway terms (Figure 3D, Supplementary Table 12) were related to cellular calcium regulation (C57BL/6J_ant_: 19 gene sets), general processes involved in the development and function of the nervous system (C57BL/6J_post_: 594 gene sets), pore formation and inflammasome activity (B6D2F1N_ant_: 37 gene sets), and nerve growth factor activity promoting neuronal differentiation and survival (B6D2F1N_post_: 39 gene sets).

**Table 4:** Top 10 differentially expressed genes between the enriched and standard housing conditions in the anterior and posterior portions of the amygdala in C57BL/6J and B6D2F1N mouse strains.

Strain	Anterior amygdala				Posterior amygdala			
	Gene	LFC	SE	adj.p	Gene	LFC	SE	adj.p
C57BL/6J genotype	*Unc13c*	2.172	0.389	4.03E-04	*Mbp*	1.960	0.246	3.10E-11
	*Phb2*	−1.684	0.318	1.03E-03	*9130024F11Rik*	3.950	0.502	3.67E-11
	*Ccdc85b*	−3.678	0.706	1.11E-03	*Padi2*	2.855	0.387	1.08E-09
	*2610028E06Rik*	7.792	1.543	1.95E-03	*Trp53inp2*	1.507	0.228	1.75E-07
	*Adcy8*	1.998	0.406	2.14E-03	*Chrna6*	9.038	1.379	2.24E-07
	*Tmem230*	−1.521	0.309	2.14E-03	*Plin4*	1.553	0.255	3.58E-06
	*Gm37943*	7.383	1.482	2.14E-03	*Csrp1*	2.670	0.440	3.58E-06
	*A830019L24Rik*	−5.374	1.097	2.14E-03	*Mobp*	1.807	0.299	3.58E-06
	*Rps19bp1*	−1.604	0.335	3.07E-03	*Mag*	1.524	0.253	3.86E-06
	*Gm30698*	−7.711	1.608	3.07E-03	*Lgi3*	1.698	0.292	1.15E-05
B6D2F1N genotype	*Gm42944*	−7.613	1.203	4.87E-06	*Mpv17l*	−0.954	0.167	2.49E-04
	*Gm10384*	8.062	1.513	9.98E-04	*Rreb1*	1.301	0.255	3.55E-03
	*Gsdmd*	−4.578	0.900	1.82E-03	*St6gal2*	−1.136	0.236	1.06E-02
	*Cuzd1*	7.308	1.434	1.82E-03	*Prkce*	−0.619	0.134	1.77E-02
	*Tenm3*	1.161	0.260	3.10E-02	*Ntf3*	−2.099	0.455	1.77E-02
	*Gm43019*	−6.042	1.392	4.74E-02	*Cib2*	−1.554	0.344	2.27E-02
					*Rprm*	−1.738	0.397	3.69E-02
					*Tnn*	6.828	1.619	4.52E-02
					*Zfp827*	−1.092	0.259	4.52E-02
					*Kank4os*	6.233	1.473	4.52E-02

*LFC, log2 fold change; SE, standard error; adj.p, Benjamini-Hochberg-adjusted *p*-value*.

### Effects of cognitive bias on the amygdala’s transcriptome

Finally, when comparing mice showing the highest and lowest optimism scores, we found 19 genes that were dysregulated in each portion of the amygdala (Table 5). One of these genes, the long non-coding RNA *Xist*, was strongly upregulated in both anterior and posterior portions of the amygdala in mice with a positive bias (“optimistic” mice), compared to mice with a negative bias (“pessimistic” mice). Despite the lack of overrepresentation in functional gene sets, the majority of the dysregulated genes obtained in these analyses appear to participate in the regulation of some sort of molecular/cellular process, from transcription (e.g., *Zfp551*, *Zfp870*, *Mettl4*) to metabolism (e.g., *Cdkl3*, *Ispd*/*Crppa*), immunity (e.g., *Fgl2*, *Arhgap19*, *Prkcq*, *C1ql2*, *Dusp10*), and differentiation (e.g., *Fam101b*, *Gpr149*).

**Table 5:** Differentially expressed genes between mice with optimistic and pessimistic cognitive biases.

Anterior amygdala			Posterior amygdala		
Gene	LFC	adj.p	Gene	LFC	adj.p
*Xist*	13.149	2.91E-30	*Xist*	13.767	7.09E-22
*Zfp937*	6.565	1.00E-03	*C230085N15Rik*	9.585	1.37E-05
*Zfp551*	9.177	1.09E-03	*Ispd*	4.274	2.32E-05
*Gm39822*	10.007	1.13E-03	*Zfp870*	9.161	7.89E-04
*Kcnu1*	8.865	1.15E-02	*Gm44433*	9.094	2.30E-03
*Arhgap19*	9.005	1.56E-02	*Gpr149*	9.806	6.64E-03
*Cdkl3*	5.054	1.97E-02	*Hacl1*	3.850	1.31E-02
*Ccdc103*	8.765	1.97E-02	*Prkcq*	6.042	1.95E-02
*Fam101b*	3.829	2.32E-02	*Jrk*	8.603	1.95E-02
*Fgl2*	4.104	2.95E-02	*Slc12a8*	8.756	2.29E-02
*Gm36529*	8.680	4.34E-02	*Fam35a*	6.251	2.96E-02
*Fam159b*	8.608	4.41E-02	*Zfp438*	4.371	2.96E-02
*Sertad4*	5.564	4.77E-02	*Exph5*	5.456	4.89E-02
*Brat1*	−1.890	4.93E-02	*C1ql2*	−2.536	4.89E-02
*Ccdc51*	4.677	4.93E-02	*Dusp10*	8.555	4.89E-02
*Npas4*	8.746	4.93E-02	*Mettl4*	3.550	4.89E-02
*4930539E08Rik*	6.756	4.93E-02	*Ccdc18*	6.063	4.89E-02
*Capn11*	−5.760	4.93E-02	*Rsl1*	8.324	4.89E-02
*Gm47483*	5.064	4.93E-02	*Gm12056*	−7.231	4.89E-02

*LFC, log2 fold change; adj.p, Benjamini-Hochberg-adjusted *p*-value*.

## Discussion

We used mice with two different genetic backgrounds, housed in standard or enriched conditions to study the effects of genetics and different environments on the amygdala’s transcriptome (as a proxy of function), and the amygdala’s role in emotional cognitive bias. We observed wide transcriptional differences related to the genetic background and anatomical localization in the amygdala of these mice. Contrary to our expectation, however, no association between the calculated optimism scores and genotype or environmental conditions was found, and the expression of only a few genes in the amygdala could be linked to these scores in our study.

Given the anatomy and physiology of the diverse nuclei composing the amygdala (Smail et al., 2020), we can expect that the anterior and posterior portions diverge in functionality and that this is reflected in their respective transcriptional patterns. Therefore, we hypothesized that genetic and environmental differences between the mice in our study might differentially impact the anterior and posterior amygdala transcriptomes. Here, we consistently observed more changes in the posterior amygdala with respect to the anterior portion, which we believe might derive from a greater role of the amygdala-hippocampal circuitry in learning and memory processes (Yang and Wang, 2017), such as those required by the CJB testing paradigm. Interestingly, though, strain-specific differences between both portions of the amygdala focused on stress and immune pathways, indicating that these mouse strains may respond differently to environmental stimuli. Indeed, this is in line with our observations of the effects of different environmental (housing) conditions, where we found wider transcriptional responses to the enriched environment in C57BL/6J mice than in the mice of the B6D2F1N strain. Nonetheless, these differences largely implicated processes involved in neurogenesis and neurotransmission in both strains. These findings are in accordance with the literature, as it has been established that environmental enrichment positively affects synaptic plasticity and activity, neurogenesis, and dentritic arborization (in the amygdala, only after chronic stress), resulting in improved immunity, stress coping abilities, mood, and behavior, among other beneficial effects (Hintiryan et al., 2021).

Because large genome-wide association studies have proven that psychiatric diseases that impact higher cortical functions, including mood, perception, and behavior, have a relatively strong genetic component as well as a highly polygenic and pleiotropic genetic architecture (Smoller, 2016; Sullivan and Geschwind, 2019; Lee et al., 2021), we were interested in assessing the effects of the genomic background on the amygdala’s function. We used the inbred C57BL/6J and hybrid B6D2F1N mouse strains because animals of these strains are visually indistinguishable, thus blinding of the experiments was possible. Importantly, these strains possess a close genetic distance. However, similar to humans, mice with closely related genetic backgrounds can present considerable phenotypic variation. The study of this variation has been particularly focused on behavioral phenotypes, such as responses to exercise training, open field activity, fear conditioning, memory, and learning (Bothe et al., 2005; Massett and Berk, 2005; Eltokhi et al., 2020). Meanwhile, studies of strain-dependent variation in biological (endo)phenotypes, including immune responses and tissue-specific gene expression (Keane et al., 2011; Sellers, 2017), are relatively limited. In this context, our study adds to the evidence of strain-dependent phenotypical variation by showing that strain-specific patterns of gene expression and responses to the environment exist even within the same laboratory and handling protocols. Moreover, differences in the amygdala’s transcriptome between strains were also related, to a great extent, to immune and stress responses. This further supports the documented immune phenotypic variation between mouse strains (Sellers, 2017), and the link between behavior and cognition with immunity (Miller et al., 2017).

Although we had a major interest in identifying the molecular correlates of emotional cognitive bias and of the effects of genetics and environmental enrichment on optimism levels, we found only a few associations in the amygdala that pointed towards differences in metabolic, immune, and developmental pathways between animals with positive and negative biases. Interestingly, we also found at least some evidence of a potential correlation between cognitive bias and lipid metabolism in the anterior amygdala. Finding no differences in optimism scores between mouse strains or housing environments was unexpected for us. However, a number of reasons may contribute to this: (a) the difference between standard and enriched environmental conditions was not very pronounced in our settings. Therefore, it is possible that a longer exposure to the playgrounds and/or a comparison against an impoverished or stressful environment would be required to achieve a significant effect; (b) as our mice were not manipulated to model a specific type of disorder associated with emotional cognitive bias (e.g., depression), we might require a larger sample size to observe differences in such behaviors; (c) the timing in our experiments might have been suboptimal for either sampling of the amygdala (cognitive bias during testing might differ from that shown during amygdala sampling) and/or evoking a bias shift (we might have missed sensitive developmental periods for the induction of emotional cognitive biases); (d) moreover, we used only female mice in our experiments. Previous research has suggested that negative cognitive bias is more common and severe in women than in men, which could explain the bias toward negative optimism scores in our sample. This disparity contributes to sex-specific differences in the onset, maintenance/relapse, and severity of human depression. However, in rats, it has been reported that negative cognitive bias is greater in middle-aged male animals than in middle-aged females, as well as in adult compared to young rats. The study also showed that the accompanying activation of the amygdala and other brain regions involved (e.g., hippocampus, nucleus accumbens, frontal cortex) are age- and sex-specific (Hodges et al., 2022).

Another limitation of our study for the exploration of the molecular correlates of cognitive bias is that we had an insufficient number of mice to apply the extremes approach to each experimental group. This made it impossible to test the effects of genetic background and/or environmental enrichment on the transcriptional patterns in the amygdala according to the animals’ emotional cognitive bias. Studies have shown that genetic variants can enhance sensitivity to the environment. This, in turn, increases the risk of psychiatric diseases such as depression and anxiety (Fox and Beevers, 2016). Taken together, we suggest that future molecular studies of emotional cognitive bias should explore age- and sex-specific transcriptional responses in limbic as well as hippocampal and frontal networks using animals from different genetic backgrounds that are exposed to enriched as well as stressful environments. This would advance our understanding of the neurobiology of negative bias and potentially of its relationship with mood disorders.

Despite the aforementioned limitations, we have been successful at extracting some promising hints regarding the biological pathways involved in emotional cognitive bias in the amygdala. In alignment with our findings, which implicate metabolic pathways (particularly of lipids) as well as immune processes, previous research has shown that optimism is associated with a higher diet quality, higher HDL cholesterol, lower triglycerides, lower risk of cardiovascular diseases, improved cell-mediated immunity, and lower production of pro-inflammatory mediators (Segerstrom and Sephton, 2010; Boehm et al., 2013; Curzytek et al., 2018; Ait-Hadad et al., 2020; Krittanawong et al., 2022).

## Conclusions

We found important differences in the transcriptional patterns in the amygdala that were associated with the animals’ genetic background but not with the animals’ cognitive bias. We also demonstrated that differences in the transcriptional response to the housing environment are inherent to the animals’ genotypes. With this, our study underscores the importance of appropriate selection of mouse strains when performing molecular studies of affective behavior, and points to a major issue affecting replicability and translational impact of such studies. Moreover, our findings hint at the involvement of lipid metabolism in optimistic cognitive bias and, overall, suggest a crucial role of immunity in the function of the amygdala and, therefore, potentially as well in emotional processing.

## Data Availability Statement

The datasets presented in this study can be found in online repositories. The names of the repository/repositories and accession number(s) can be found below: Raw and processed RNA-seq data has been deposited into NCBI’s Gene Expression Omnibus (GEO) database and is accessible with the ID GSE209999.

## Ethics Statement

The animal study was reviewed and approved by Gesundheits- und Veterinäramt Münster, Nordrhein-Westfalen (reference number: 39.32.7.1) and Landesamt für Natur, Umwelt und Verbraucherschutz Nordrhein-Westfalen (“LANUV NRW”).

## Author Contributions

SK, SR, MS, and NS conceived the study. LB, SK, SR, NS, and MS designed the experiments. LB carried out the experimental procedures and sample collection, and prepared the manuscript and Figure 1. MH-R designed the analysis strategy, processed and analyzed the data, and prepared the manuscript, figures and tables. AW supervised sequencing. All authors contributed to the article and approved the submitted version.

## Funding

This research was funded by the German Research Foundation (Deutsche Forschungsgemeinschaft, DFG) as part of the SFB TRR 212 (NC^3^), project numbers 316099922 and 396776123. Principal investigators: SR and NS.

## Conflict of Interest

The authors declare that the research was conducted in the absence of any commercial or financial relationships that could be construed as a potential conflict of interest.

## Publisher’s Note

All claims expressed in this article are solely those of the authors and do not necessarily represent those of their affiliated organizations, or those of the publisher, the editors and the reviewers. Any product that may be evaluated in this article, or claim that may be made by its manufacturer, is not guaranteed or endorsed by the publisher.

## Abbreviations

TS: touch screen
CJB: cognitive judgement bias
GO: gene ontology
BP: biological processes
MF: molecular function
CC: cellular component
KEGG: Kyoto Encyclopedia of Genes and Genomes
DEG: differentially expressed gene.
